# An Obstacle Detection Method Based on Longitudinal Active Vision

**DOI:** 10.3390/s24134407

**Published:** 2024-07-07

**Authors:** Shuyue Shi, Juan Ni, Xiangcun Kong, Huajian Zhu, Jiaze Zhan, Qintao Sun, Yi Xu

**Affiliations:** 1 School of Transportation and Vehicle Engineering, Shandong University of Technology, Zibo 255000, China; 22502060007@stumail.sdut.edu.cn (S.S.); 22502060003@stumail.sdut.edu.cn (J.N.); 22502060006@stumail.sdut.edu.cn (X.K.); 23402030186@stumail.sdut.edu.cn (H.Z.); 23502060271@stumail.sdut.edu.cn (J.Z.); 23402030185@stumail.sdut.edu.cn (Q.S.); 2 Qingte Group Co., Ltd., Qingdao 266106, China

**Keywords:** longitudinal active vision, image processing, distance estimation, camera rotation strategy

## Abstract

The types of obstacles encountered in the road environment are complex and diverse, and accurate and reliable detection of obstacles is the key to improving traffic safety. Traditional obstacle detection methods are limited by the type of samples and therefore cannot detect others comprehensively. Therefore, this paper proposes an obstacle detection method based on longitudinal active vision. The obstacles are recognized according to the height difference characteristics between the obstacle imaging points and the ground points in the image, and the obstacle detection in the target area is realized without accurately distinguishing the obstacle categories, which reduces the spatial and temporal complexity of the road environment perception. The method of this paper is compared and analyzed with the obstacle detection methods based on VIDAR (vision-IMU based detection and range method), VIDAR + MSER, and YOLOv8s. The experimental results show that the method in this paper has high detection accuracy and verifies the feasibility of obstacle detection in road environments where unknown obstacles exist.

## 1. Introduction

With the improvement in people’s living standards and the progress of science and technology, automobiles have become the most common means of transportation in modern travel. With the increase in the number of automobiles in countries all over the world, the numbers of related automobile safety problems have also risen. According to the World Health Organization, the number of road traffic deaths has decreased by 5% per year since 2010, down to 1.19 million per year [1]. However, road traffic crashes remain a persistent global health crisis, with pedestrians, cyclists and other vulnerable road users facing a serious and rising risk of death. In order to improve road traffic safety, research in the field of automotive safety has evolved towards smarter Advanced Driving Assistance Systems (ADASs) [2]. An ADAS is the use of environmental sensing technology to collect dynamic data from the vehicle, driver and surrounding environment and analyze and process them, in order to achieve safe and comfortable driving and minimize traffic accidents by alerting the driver or actuators to intervene in the vehicle’s maneuvering. In addition, it also provides reliable safety support for FSD (full self-driving). Therefore, obstacle detection is an important element in the structure of intelligent-vehicle environment-sensing technology, and it is also a prerequisite for realizing advanced assisted-driving functions.

The complex road environment brings great difficulties for the intelligent vehicle’s environment perception; the reliable and accurate detection of obstacles is one of the core problems that need to be solved to realize automobile assisted driving [3]. In addition to the innovation of algorithms, developments in technology have made great progress in obstacle detection, and sensor types, target objects, and detection methods have become the main research content. The sensor aspect includes cameras [4], radar [5,6], etc. Camera detection technology is widely used in obstacle detection due to its relative maturity and low cost. In this paper, a novel obstacle detection method is proposed, which uses a monocular camera as a visual sensor and servo motor to form a longitudinal active camera detection system to detect various types of obstacles in front of the vehicle in the road environment, including vehicles, pedestrians, and conical obstacles, spherical obstacles, rod obstacles, cartons and so on. The principle of the detection method in this paper is to use the two frames of images acquired before and after the camera rotates along the longitudinal plane as the detection object, use the height difference characteristics of the obstacle point and the ground point to detect the obstacle, obtain the distance information between the obstacle and the vehicle, and realize the detection of the obstacle in front of the vehicle.

The rest of the paper is organized as follows: Section 2 describes the existing research methods. Section 3 presents the principles of an obstacle detection method based on longitudinal active vision, including the detection process, obstacle extraction algorithms based on fast image region matching in stable extreme regions (MSER), monocular ranging models, and static and dynamic obstacle detection models. Section 4 designs the obstacle detection experiments for the theoretical method proposed in this paper, compares the obstacle detection methods based on YOLOv8s and VIDAR, and verifies the superiority of the method in this paper. Section 5 summarizes the content of this paper.

## 2. Related Work

Obstacle detection is a core component of automatic driving technology, which is crucial for promoting the development of automatic driving technology and has become a key area of research in recent years. Monocular vision sensors collect rich information, with strong applicability and a low price; many scholars have conducted relevant research on this, mainly divided into two kinds of obstacle detection algorithms, either based on image features or based on deep learning. With the rapid development of computer technology, deep learning is increasingly used in target detection. Target detection algorithms based on deep learning use convolutional neural networks (CNN) to learn features; this feature learning method is mainly through input training set to automatically learn the feature information of the target, and the neural network to generate more advanced, more abstract and more reliable feature information. Han et al. [7] proposed CNN-based real-time semantic segmentation of class-aware edge information based on channel-aware attention and constructed a new EdgeNet network with significant improvement in MIOU compared to other networks. Nguyen [8] proposed an improved framework based on a fast, reactive neural network using MobileNet architecture to build the base convolutional layer of fast R-CNN, which utilizes the deeply divisible convolutional structure in the mobile network architecture to build the classifier and improve the accuracy of vehicle detection. Zaghari et al. [9] used the YOLO non-maximum suppression (NMS) algorithm for obstacle detection, which has higher detection accuracy and faster detection speed compared to other algorithms. Yasmin et al. [10] proposed an approach based on transfer learning to detect small obstacles under strict lighting and illumination conditions using UNet++, PSPNet, PANNet, LinkNet and DeepLabV3+ semantic segmentation models. He et al. [11] proposed a flexible and efficient multi-scale unipolar target detector FE-YOLO for image obstacle detection. Sun et al. [12] proposed a real-time fused semantic segmentation network RFNet, which can effectively utilize depth complementary features, multi-dataset training and depth streaming in the architecture makes the network very effective in detecting unexpected small targets. However, the RFNet network performs poorly in detecting features such as sidewalks, walls, and motorcycles.

Detecting obstacles using deep learning methods requires prior knowledge about the obstacle; therefore, obstacle detection methods based on deep learning have some limitations. In obstacle detection based on conventional image features, GÜNGÖR et al. [13] proposed a new non-artificial intelligence method for finding ground lines and detecting obstacles on the road using v-parallax data. Yuan et al. [14] used U–V parallax images to detect the drivable area of an unmanned vehicle using U–V parallax images as well as the geometric relationship between the size of an obstacle and its parallax to detect obstacles in the drivable area. Xu et al. [15] proposed a generalized obstacle detection method based on VIDAR combined with a fast image region-matching method based on MSER with high detection accuracy. Kumar et al. [16] accomplished the identification of defective regions on the surface of an obstacle by means of a multilevel color threshold segmentation method. Wang et al. [17] used a single wide-angle camera for real-time obstacle detection, based on the background difference method for detecting static and moving objects. Xue et al. [18] proposed a method for detecting obstacles on a reflective ground, using a pre-calibrated ground-based detection scheme that characterizes the difference between obstacles and non-obstacles, and locates obstacles by means of appearance–geometry fusion models. Optical flow methods can detect moving obstacle targets based on changes in the image optical flow field caused by object motion, with good robustness and without the need to know a priori information about the obstacle. Therefore, optical flow methods have been widely studied and applied [19,20,21].

However, today’s research techniques still contain deficiencies. The obstacle detection method based on VIDAR, although highly accurate, is limited in usage scenarios, and their applicability decreases when vehicles are stationary or at low speeds. Deep learning is limited by the sample type and cannot detect obstacles of unknown shape. Therefore, this paper proposes an obstacle detection method based on longitudinal active vision, which does not require a priori knowledge of the scene to achieve more accurate obstacle recognition and distance detection.

## 3. Methods

**Figure 1:**
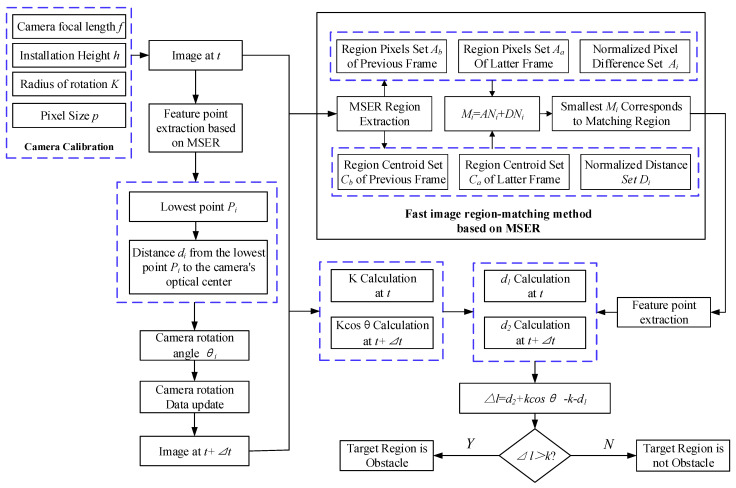
An obstacle detection based on longitudinal active vision.

In this paper, we combine a fast image region-matching method based on MSER with an obstacle detection method based on longitudinal active vision to simplify the matching process, improve the matching speed, and use fewer feature points for obstacle detection. The process of the road obstacle detection method based on longitudinal active vision studied in this paper is as follows and is shown in Figure 1.

(1) Calibration of initial camera parameters

Calibrate the initial internal and external parameters of the camera mounted on the vehicle, obtain the camera’s focal length f by camera calibration, the pixel size p of the photosensitive chip, obtain the camera mounting height h by measurement calculation, and the camera rotation radius K.

(2) Image region matching and obstacle detection
  ① Acquire the initial frame image. At the moment t = 0, the camera obtains the first frame image $I_{ft}$. The feature points in $I_{ft}$ are extracted, and the lowest point of the extracted feature points is considered as the intersection point $P_{i}$ of the obstacle and the road plane. The distance $d_{i}$ from point $P_{i}$ to the camera is then calculated, resulting in the required rotation angle $\theta_{i}$ for the camera to obtain the next frame image.
  ② Acquire the second frame image. The second frame image $I_{bt}$ is acquired after the camera is rotated by an angle $\theta_{i}$. The fast image region-matching method based on MSER is performed on $I_{ft}$ and $I_{bt}$ to find the center of mass of the matched region as feature points.
  ③ Calculate the horizontal distance from the camera to the center of rotation. Calculate the horizontal distance Kcosθ from the camera to the center of rotation based on the camera rotation angle $\theta_{i}$ and camera rotation radius K obtained in ①.
  ④ Horizontal distance calculation. Assuming that the feature point is on the horizontal plane, the horizontal distance $d_{1}$, $d_{2}$ from the feature point to the camera is calculated based on the monocular ranging model at the before and after moments, respectively.
  ⑤ Obstacle judgment. According to the internal and external parameters of the camera as well as the acquired valid information, compare Δl ($\Delta l=d_{2}+K\cos\theta -d_{1}-K$) and k (k is a set threshold, k>0). The feature point is not on the horizontal plane and the region is an obstacle if Δl>k. The feature point is on the horizontal plane and the region is not an obstacle if Δl≤k.

(3) Camera reset

After completing the two-frame image detection, the camera resets and repeats step (2) to proceed to the next stage of active obstacle detection.

### 3.1. Fast Image Region-Matching Method Based on MSER

The MSER algorithm is a region-based feature-extraction algorithm that is widely used in image matching and target tracking [22,23,24,25]. Since the detection object of this method is the region features of the image rather than the local features, the detected feature points are more stable, and the obstacles in the image can be detected quickly and the non-obstacles can be eliminated. The fast image region-matching method based on MSER ignores the position and shape differences of the MSER algorithm between two images, and the specific matching method process is as follows:

(1) Extract the region of maximum stable extremes using the MSER algorithm.
(2) Perform region range difference $AN_{i}$ calculation. For the two frames captured in the experiment, it is assumed that the MSER region sets of the two frames before and after are $Af=\left\{Af_{1},Af_{2},\cdots ,Af_{n}\right\}$ and $As=\left\{As_{1},As_{2},\cdots ,As_{n}\right\}$, respectively. $A_{i}$ is the set of the difference between the *f*th MSER region range in the previous frame and the unmatched region in the next frame. The set $A_{i}$ is normalized and the effect of normalization is denoted by $AN_{i}$, where
  (1) $$AN_{i}=\frac{Ai-\min(Ai)}{\max(Ai)-\min(Ai)}$$
(3) Perform region set spacing $DN_{j}$ calculation. It is assumed that the MSER region center-of-mass sets in the front and back images are $Df=\left\{Df_{1},Df_{2},\cdots ,Df_{m}\right\}$ and $Ds=\left\{Ds_{1},Ds_{2},\cdots ,Ds_{m}\right\}$, respectively. $D_{j}$ is the set of distances between the *f*th MSER region range in the previous image and the unmatched region in the latter image. The set $D_{j}$ is normalized and the result is denoted by $DN_{j}$, where
  (2) $$DN_{j}=\frac{D_{j}-\min(D_{j})}{\max(D_{j})-\min(D_{j})}$$
(4) Extract the matching region $M_{l}$. Let $M_{l}$ be the set of matching values of the *s* th MSER, and extract the MSER corresponding to the smallest $M_{l}$ as a matching region, denoted as $M_{l}=AN_{i}+DN_{j}$.

Using the stabilized feature points extracted using the algorithm based on MSER for obstacle detection can shorten the post-processing image time and improve the speed of obstacle detection.

### 3.2. Static Obstacle Detection Model

**Figure 2:**
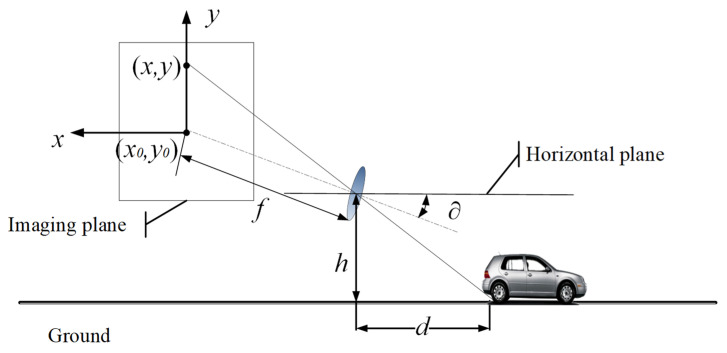
Obstacle ranging model.

Measurement methods based on monocular vision need to obtain position information in three-dimensional space from two-dimensional image information, and the obstacle ranging principle can be described by the pinhole model principle, which can accurately calculate the distance between the vehicle and the obstacle. As shown in Figure 2, a camera is loaded on the auto-vehicle, the object in front of the auto-vehicle is regarded as an obstacle, and the lowest point of the obstacle is regarded as the intersection of the obstacle and the road surface.

Here, the focal length of the camera is f and the pixel is μ. The pitch angle of the camera installation is ∂ and the height of the installation is h. Let $\left(x_{0},y_{0}\right)$ be the coordinate origin in the image plane and (x,y) be the coordinates of the intersection of the obstacle and the road plane in the imaging plane. Then the horizontal distance between the camera and the obstacle can be found by Equation (3).

(3) $$d=\frac{h}{\tan\left(\partial +\arctan\left[\left(y_{0}-y\right)\mu /f\right]\right)}$$

**Figure 3:**
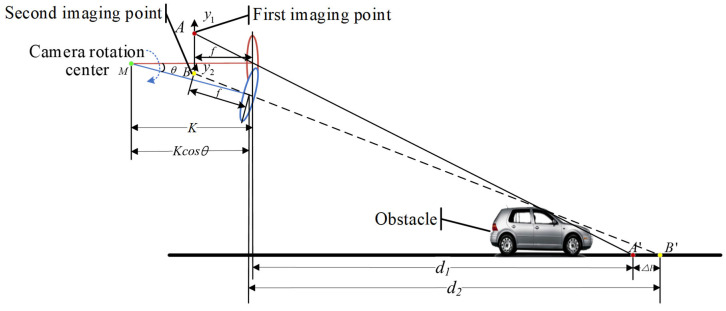
Schematic diagram of the static obstacle imaging.

As shown in Figure 3, the first imaging point of the vertex of the obstacle at the initial moment is A. At the next moment, the y-axis rotates and translates from the image plane from $y_{1}$ to $y_{2}$ due to the camera making a longitudinal rotation in the plane with the point M as the center of rotation, and the second imaging point of the vertex of the obstacle is B. The horizontal distance from the camera optical center to the point M is K in the previous frame, and the horizontal distance from the optical center to the point M is Kcosθ after the camera is rotated by an angle θ. $A^{\prime }$ and $B^{\prime }$ are points on the road plane projected from the vertex of the obstacle, and Δl is the horizontal distance between the two projected points. The horizontal distance from the camera optical center to $A^{\prime }$ is $d_{1}$, and the horizontal distance to $B^{\prime }$ is $d_{2}$. $d_{1}$ and $d_{2}$ can be calculated by Equation (3). $d_{1}$ and $d_{2}$ are related by the equation $d_{1}+K+\Delta l=d_{2}+K\cos\theta$. If the feature point is a point on the ground, the equation for $d_{1}$ and $d_{2}$ should be $d_{1}+K=d_{2}+K\cos\theta$. If $d_{1}+K\neq d_{2}+K\cos\theta$, it means that the target point is a point with height and therefore the obstacle can be recognized by Δl. Figure 3 shows the theoretical model for detecting static obstacles when the vehicle is stationary, and the displacement in the horizontal direction due to camera rotation is K−Kcosθ. And when the vehicle is in motion, the actual displacement of the camera in the horizontal direction is Δd. Δd can be calculated by Equation (5). Δl can be calculated by Equation (6).

(4) $$t_{s}=\frac{\theta }{v_{s}}$$

(5) $$\Delta d=t_{s}\times v_{c}-K+K\cos\theta$$

(6) $$\begin{array}{l} \Delta l=d_{2}+\Delta d-d_{1}\\ =d_{2}+\frac{\theta \times v_{c}}{v_{s}}-K+K\cos\theta -d_{1} \end{array}$$

where $t_{s}$ is the time to acquire two frames of image camera rotation, $v_{s}$ is the speed of the servo motor, and $v_{c}$ is the vehicle speed.

### 3.3. Dynamic Obstacle Detection Model

**Figure 4:**
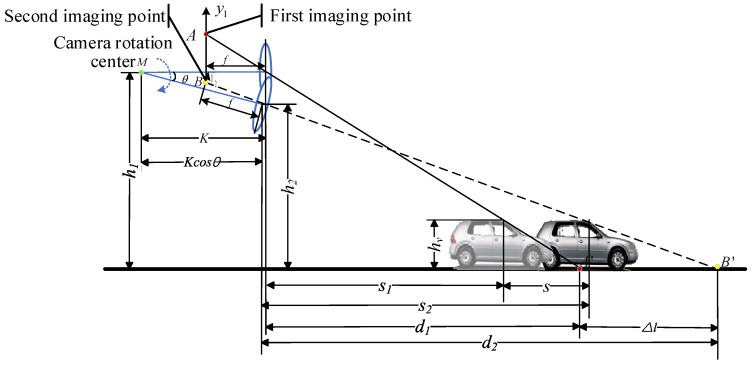
Schematic diagram of dynamic obstacle imaging.

When the obstacle in front of the vehicle moves in the horizontal direction, the imaging of the moving obstacle is shown in Figure 4. The camera pitch angle at the initial moment is 0. Therefore, the distance from the camera rotation center point M to the ground is equal to the distance from the camera optical center to the ground, which is $h_{1}$. The distance between the optical center and the ground after the camera is rotated by an angle θ is $h_{2}$. The horizontal distance between the optical center of the camera and the vertex of the obstacle at the initial moment is $s_{1}$. The horizontal distance between the optical center and the vertex of the obstacle after the camera is rotated by an angle θ is $s_{2}$. The distance moved by the obstacle is s. When the target obstacle is not moving straight, s is the distance that the actual moving distance of the obstacle projects onto the direction of the vehicle’s movement. Then the relationship between $d_{1}$, $d_{2}$, $s_{1}$, $s_{2}$ and s is:

(7) $$\{\begin{array}{c} K\cos\theta +d_{2}=K+d_{1}+\Delta l\\ K-K\cos\theta +s_{1}+s=s_{2} \end{array}$$

The height of the obstacle is $h_{v}$. According to the principles of a right triangle, the relationship between $h_{v}$, $h_{1}$, $h_{2}$, $d_{1}$, $d_{2}$, $s_{1}$ and $s_{2}$ can be expressed as:

(8) $$\{\begin{array}{c} \frac{h_{v}}{h_{1}}=\frac{d_{1}-s_{1}}{d_{1}}\\ \frac{h_{v}}{h_{2}}=\frac{d_{2}-s_{2}}{d_{2}} \end{array}$$

From Equations (4) and (5):

(9) $$\Delta l=\frac{\left(h_{1}-h_{v})(sh_{2}-sh_{v}+s_{2}h_{v})-s_{1}h_{v}(h_{2}-h_{v}\right)}{\left(h_{1}-h_{v})(h_{2}-h_{v}\right)}$$

Dynamic obstacles can be recognized when $\left(h_{1}-h_{v})(sh_{2}-sh_{v}+s_{2}h_{v})\neq s_{1}h_{v}(h_{2}-h_{v}\right)$. Thus obstacles on the road can be recognized using camera rotation. The judgment process only requires tracking of the feature points, calculating their positions, and obtaining the height of the optical center above the ground after camera rotation, thus reducing the consumption of time and space for obstacle detection. From Equation (9), it can be seen that the dynamic obstacle detection model is only related to the parameters such as the height above the ground before and after the movement of the camera, the moving distance of the target obstacle, and has nothing to do with the state of the self-vehicle, the camera rotating speed and the camera rotating angle, so that the vehicle in both stationary and motion state can realize the accurate detection of obstacles.

### 3.4. Camera Rotation Strategy

**Figure 5:**
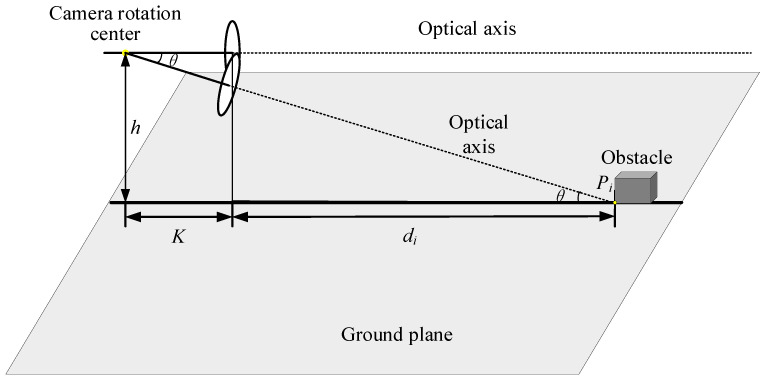
Schematic diagram of the camera rotation.

The theoretical models of static and dynamic obstacle detection presented in Section 3.2 and Section 3.3 are based on two frames of images captured before and after camera rotation, and the target always remains within the field of view before and after camera rotation. The rotation angle of the camera is calculated by pointing the optical axis to the lowest point of the obstacle based on the positional information of the obstacle. As shown in Figure 5, the obstacle is located in front of the vehicle, when the camera is located in the initial position to acquire the first frame image. Based on the MSER algorithm to obtain a stable extreme value region, the lowest point of the extreme value region as a feature point, the lowest point of the extracted feature point is regarded as the intersection point $P_{i}$ (*i* is the number of *P*-points) of the obstacle and the road plane, the optical axis points to the intersection point a at the next moment. The coordinates of point $P_{i}$ in the imaging plane are $\left(x_{i},y_{i}\right)$, the distance $d_{i}$ from the camera to $P_{i}$ can be calculated from Equation (3), and the camera’s optical axis rotation angle $\theta_{i}$ can be found based on the trigonometric relationship:

(10) $$\theta_{i}=\arctan\frac{h}{d_{i}+K}=\arctan\frac{h\tan(\arctan[(y_{0}-y_{i})\mu /f])}{h+K\tan(\arctan[(y_{0}-y_{i})\mu /f])}$$

## 4. Experiments and Results

In order to verify the feasibility of the theoretical method in this paper, simulation experiments and real-vehicle experiments under controlled scenarios are designed. The experimental results show that the obstacle detection method based on longitudinal active vision proposed in this paper can effectively detect obstacles in the road environment. By analyzing and comparing with the traditional method, it proves that the method has high detection accuracy and detection speed.

### 4.1. Experimental Equipment

**Figure 6:**
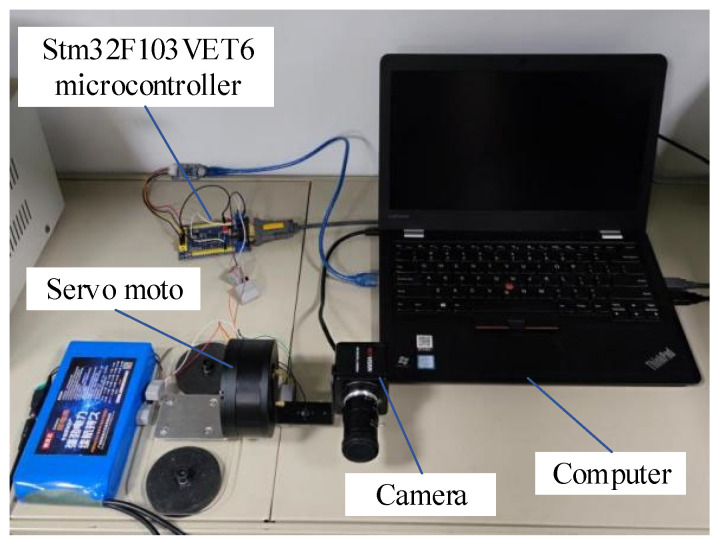
Architecture of the longitudinal active camera obstacle detection system.

In order to realize the active rotation of the camera as well as the accurate control of the camera angle and direction of rotation, this paper selects the MS9015 V1 model three-phase permanent magnet synchronous motor (Shanghai Lingkong Technology Co., Ltd., Shanghai, China), U-shaped bracket, and magnetic suction cup for the design of the active camera gimbal. The camera model selected in this paper is the RER-USB8MP02G (RERVISION Technology Co., Ltd., Shenzhen, China), which has high resolution and dynamic range, is not easily affected by temperature changes, and meets the requirements of environment sensing for the autonomous vehicle. The STM32F103VET6 microcontroller is utilized as the controller to achieve the control of the motor through voltage space-vector pulse width modulation (SVPWM). CAN communication is used between the motor and the STM32F103VET6 microcontroller (Wildfire Technology Co., Ltd., Dongguan, China) to control the camera rotation. This paper designs the longitudinal active camera obstacle detection system, as shown in Figure 6.

### 4.2. Obstacle Detection Simulation Experiment

The longitudinal active camera is mounted on the experimental platform, a vehicle scale model is used to simulate obstacles, and common non-obstacles such as traffic signs and road patches are simulated with pieces of paper attached to a flat surface, and two other unknown types of obstacles are set up. One set of obstacle simulation experiments is processed as follows.

**Figure 7:**
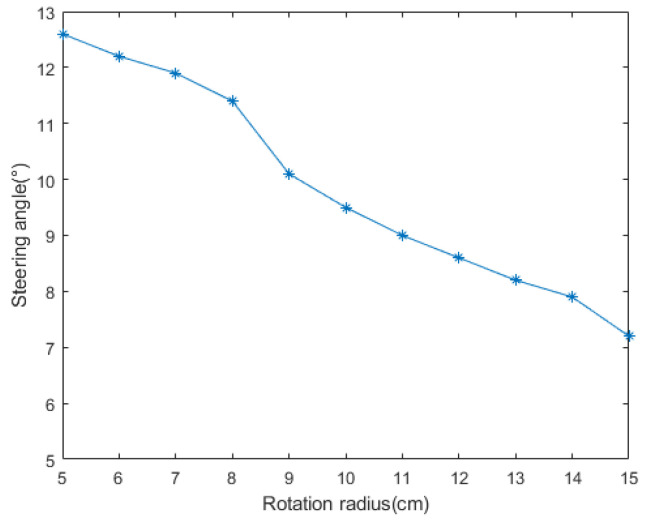
Steering angles corresponding to different radii of rotation.

**Figure 8:**
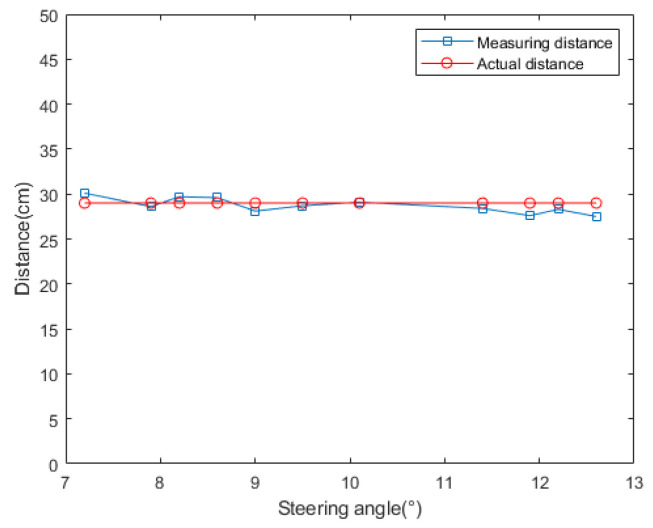
Distance measurements corresponding to the different steering angles.

**Figure 9:**
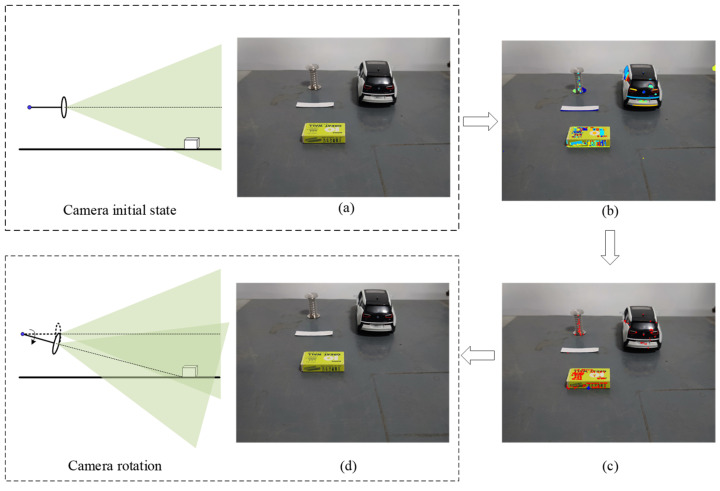
Two-frame image acquisition before and after camera rotation; (**a**) is the obstacle image at the initial moment, (**b**) is the feature region extraction based on MSER, and (**c**) is the feature point extraction, where red * is the lowest point of each extreme region and blue + is the intersection point of the obstacle and the road plane. (**d**) The second frame image acquired after camera rotation.

In the simulation experiment of obstacle detection, the camera pitch angle θ=0° at the initial moment, the effective focal length of the camera f=6.779 mm, the installation height h=18 cm. The camera rotation radius and steering angle affect the measurement accuracy of the target distance, so the corresponding camera steering angle is calculated by setting different K values, and the measured target distance is compared and analyzed with the real distance. As shown in Figure 7 and Figure 8, the steering angle decreases with the increase in the rotation radius, and the calculated camera steering angle θ=10.1° at K=9 cm, when the corresponding distance error is minimized. Therefore, the rotation radius of the camera was set to 9 cm. The camera acquires the first frame of the image at the initial moment, and detects the extreme value region in the image based on the MSER algorithm through the filtering and thresholding operation of the first frame of the image at multiple scales. According to the stability and distribution density of the region, the lowest point of the stable extreme value region is selected as the feature point, and the point with the largest value of the vertical coordinates among the various feature points under the imaging coordinate system is regarded as the intersection point of the obstacle and the road plane (Figure 9c), and the angle of rotation of the camera at the next moment θ=9° is calculated by Equation (7).

**Figure 10:**
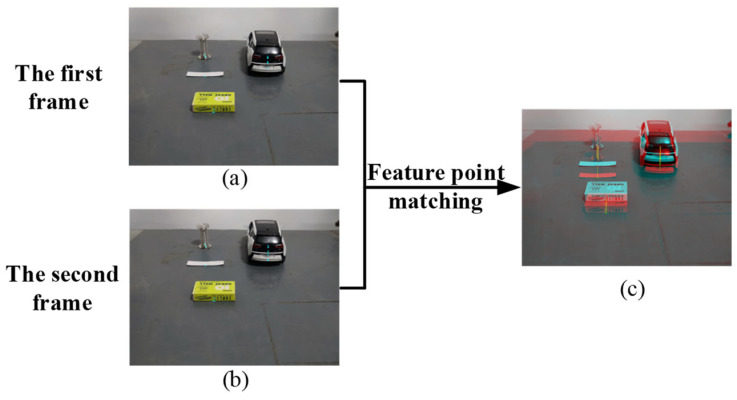
MSERs feature region extraction; (**a**) is the obstacle image in the initial moment, (**b**) the obstacle image in the next moment, and (**c**) is the region matching image in the two moments before and after, where the red region and + are the center of mass of MSERs and MSERs in the initial moment in the image, and the cyan region and o are the center of mass of MSERs and MSERs in the next moment in the image.

Based on the rotation angle calculated from the above experiments, the camera rotates to acquire the second frame image. The horizontal distances from the optical center to the center of rotation at the before and after moments were calculated by the monocular ranging model as 10 cm and 9.74 cm, respectively, and the experiments used the fast image region-matching method based on MSER to process the two frames acquired at the before and after moments. The centers of mass of the 14 extracted matching regions are used as feature points, as shown in Figure 10.

**Figure 11:**
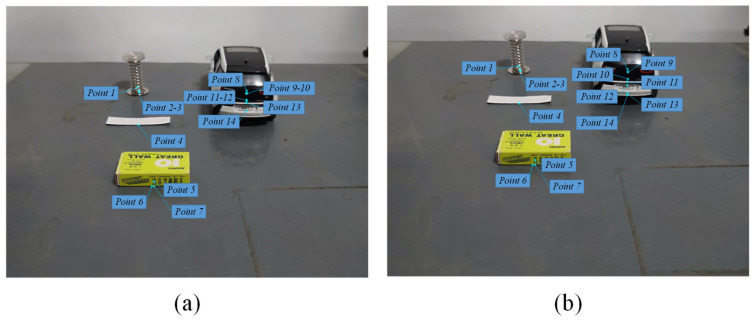
Feature point location; (**a**) shows the location of the feature point located in the image at the initial moment and (**b**) shows the location of the feature point located in the image at the next moment.

Feature point extraction is performed based on the front and rear frames acquired by the active camera (Figure 11), assuming that the feature points are on the horizontal plane, and detecting the horizontal distance $d_{1}$ and $d_{2}$ of each feature point from the camera in the front and rear frames through the monocular ranging model, and comparing Δl and k (k= 2 cm) to confirm the obstacles. $d_{1}$, $d_{2}$ and Δl are calculated as shown in Table 1.

**Figure 12:**
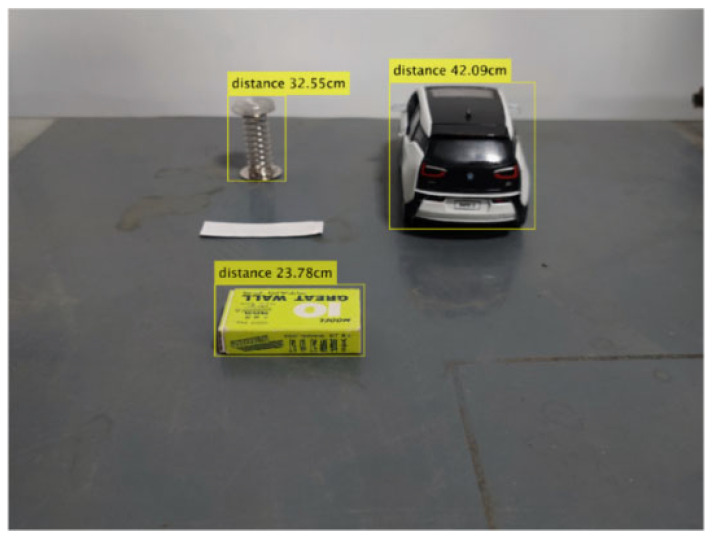
Obstacle area division (where the yellow box is the detected obstacle area and the upper number is the distance from the obstacle to the camera).

The calculation results show that the value of feature point four is 0.83 cm, which is smaller than the set threshold and is a feature point on the horizontal plane. The rest of the feature points except feature point four are not on the horizontal plane and are feature points on obstacles with height targets, and the corresponding MSERs all belong to the obstacle region. Non-obstacle points are excluded based on a set threshold, and the intersection of the bottom boundary of the obstacle region and the road plane is taken as the ranging point of the obstacle, and the distance from the obstacle to the camera is calculated using the monocular ranging model. Figure 12 shows the divided obstacle region, and the distances from the camera to the target obstacle are 42.09 cm, 23.78 cm, and 32.55 cm, respectively.

### 4.3. Obstacle Detection Real Vehicle Experiment

**Figure 13:**
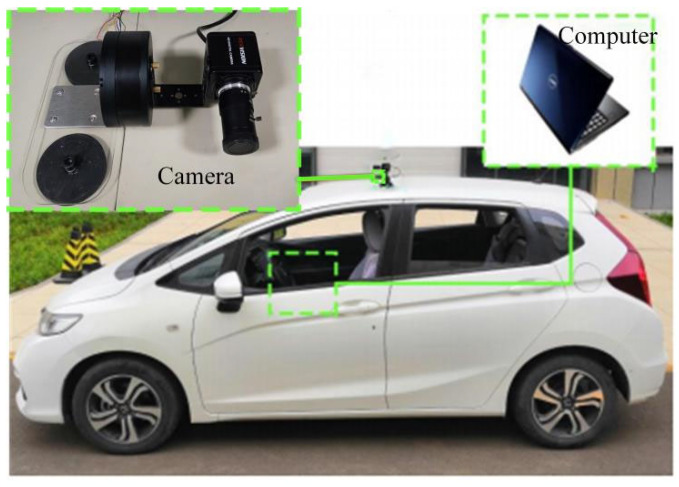
Experimental equipment for real vehicle.

**Figure 14:**
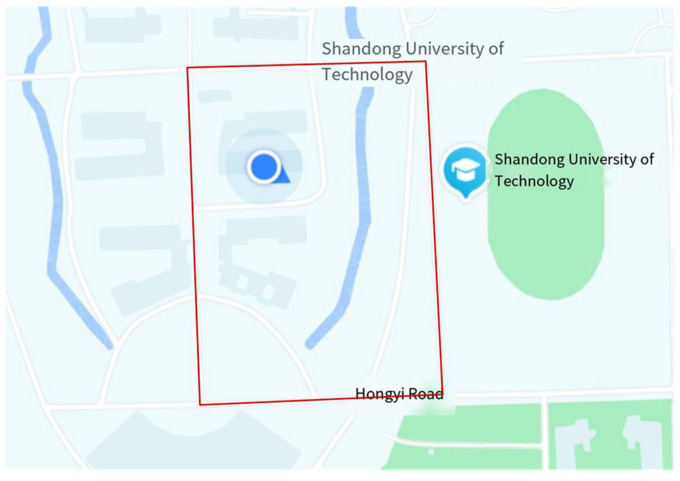
Real vehicle experiment route.

In order to verify the reliability of the method in this paper, a real vehicle experiment was set up. A Honda Fit (FIT) vehicle was used as the experiment vehicle, and a longitudinal active camera was mounted at the front position of the roof to collect environmental information, and the camera was mounted at a height of 1.60 m. The equipment for the real vehicle experiment is shown in Figure 13. A computer with an NVIDIA GeForce RTX 2080 Ti graphics card was used. It is equipped with Intel(R) Xeon(R) Silver 4210 CPU running at 2.20 GHz with 32 GB of RAM. Figure 14 shows the route of the real vehicle experiment. The experimental vehicle traveled in the campus environment at a speed of 0–30 km/h (including a straight section and a turning section), and the environmental information was collected by the camera. The total number of obstacles in the target area was 4479. The obstacles in the road were classified into two categories: 1. Pseudo obstacles with height and no risk of obstructing the vehicle’s travel. 2. Real obstacles with height and risk of obstructing the vehicle’s travel.

YOLOv8 is divided into different versions such as YOLOv8n, YOLOv8s, YOLOv8m, YOLOv8l and YOLOv8x according to the depth and width of the network. In this paper, YOLOv8s was selected for comparison experiments by taking the model size into account. The statistical data of the experimental results of four obstacle detection methods, this paper’s method, VIDAR [15], VIDAR + MSER [26], and YOLOv8s were used to measure the accuracy of the obstacle detection methods with TP, FP, TN, and FN values [27,28,29], where TP denotes that the model correctly predicts real obstacles as real obstacles, FN denotes that the model incorrectly predicts real obstacles as pseudo-obstacles, FP denotes that the model incorrectly predicts pseudo-obstacles as real obstacles, and TN denotes that the model correctly predicts pseudo-obstacles as pseudo-obstacles. The TP, FP, TN, and FN values for each method are shown in Table 2.

In the result analysis, accuracy, recall, precision, mAP were used as the evaluation index for obstacle detection methods, where mAP is the average of average precision (average precision, AP) of all categories. Accuracy indicates the proportion of correctly judged data to the total data. Recall rate for all positive cases in the data, indicating the proportion of correctly judged positive cases to all positive cases in the total data. The accuracy rate is also called the check rate, and a high accuracy rate indicates that most of the obstacles detected by the model are indeed obstacles. Since the methods in this paper, VIDAR and VIDAR + MSER are designed to categorize road obstacles into two categories, obstacles and non-obstacles and YOLOv8s contains multiple categories of obstacles, mAP is therefore used as the final evaluation metric for the four detection methods. The comparison of the detection precision of each method is shown in Table 3. Accuracy, recall and precision can be expressed, respectively, as follows:

(11) $$Accuracy=\frac{FP+FN}{TP+TN+FP+FN}$$

(12) $$Recall=\frac{FP}{TP+FP}$$

(13) $$Precision=\frac{TP}{TP+FP}$$

The results show that the mAP of the method in this paper was improved by 7.4%, 4%, and 8.6% compared to the obstacle detection methods based on VIDAR, VIDAR+MSER, and YOLOv8s, respectively. The recall improved by 3.2%, 2.4% and 4.7%. The accuracy improved by 8.3%, 6.2% and 8.6%. Compared with YOLOv8s, this paper’s method had no obvious advantage in detection time, but compared with the other two methods, this paper’s method needed to deal with fewer feature points, and the final detection time was relatively short.

**Figure 15:**
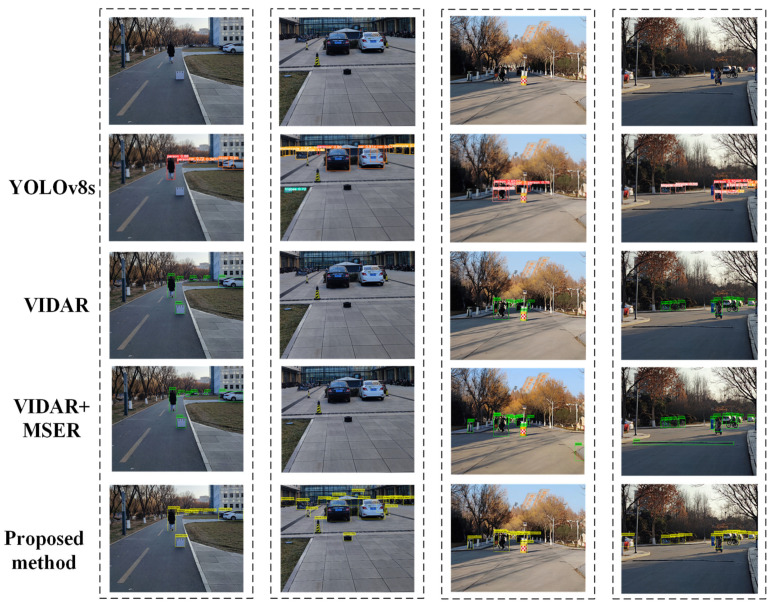
Detection results.

Figure 15 shows the obstacle detection results (partially) when the experiment vehicle is in motion and stationary. From the figure, it can be seen that the method in this paper can realize the detection of common obstacles in the road, such as vehicles, bicycles, pedestrians and so on. In addition, it can also achieve the detection of unknown types of obstacles, such as irregularly shaped tires due to breakage, road cones, barricades and so on. Poor detection is due to YOLOv8s lack of training on unknown types of obstacles. Moreover, in scenes with complex backgrounds, false detection occurs, which leads to lower detection accuracy. The second group in Figure 15 shows the images captured by the camera when the vehicle is completely stationary, and VIDAR and VIDAR + MSER cannot recognize the obstacles when the vehicle is stationary. The detection method proposed in this paper uses camera rotation to obtain image information and process the obstacle feature points. For a class of obstacles with similar color to the ground, the detection effect is poor, but the detection effect of this paper’s method is better overall than the other methods. In addition, the method used in this paper calculates the distance between the camera and the obstacle in front of it. The distance detection results of obstacles in the second set of images are shown in Table 4. By analyzing the difference between the actual distance and the measured distance, the results show that the distance error is mainly between 0.09 and 0.23. The method has an error of less than 0.15 m over short distances (<10 m); the error increases with the increase in distance, and the distance error is relatively large in the presence of obstacles that are partially obscured. From the overall results of distance measurement, the vision-based ranging algorithm proposed in this paper meets the requirements of measurement accuracy and can realize accurate distance measurement of obstacles within a short distance.

## 5. Conclusions and Future Work

In this paper, we propose an obstacle detection method based on longitudinal active vision, which realizes the detection of all types of obstacles in the road, does not depend on the accurate classification of obstacles, and reduces the spatiotemporal complexity of road environment perception. Combining the fast image region-matching method based on MSER with the obstacle detection method based on longitudinal active vision improves the speed of image matching as well as the accuracy of obstacle recognition and realizes the distance measurement of obstacles. The experimental results show that this paper’s method can effectively recognize obstacles, and the mAP of this paper’s method is improved by 7.4%, 4%, and 8.6% compared with the obstacle detection methods of VIDAR, VIDAR+MSER and YOLOv8s. The obstacle ranging error over a short distance (<10 m) is less than 0.15 m. Compared with other obstacle detection methods, the method in this paper is more applicable and can meet the detection requirements in complex environments.

In this paper, we produced a methodological innovation in obstacle detection, using the obstacle detection method based on longitudinal active vision to realize the accurate detection of road obstacles, and there are still some shortcomings. Compared with other traditional obstacle detection methods, the detection speed of this paper’s method is low and has no obvious advantages. The next step is to consider using more complex feature descriptors and matching algorithms to improve the detection speed. In addition, machine learning is being considered for combining with this paper’s model in subsequent research to realize more comprehensive and efficient obstacle detection on the roads.

## Figures and Tables

**Table 1:** Calculation results of $d_{1}$, $d_{2}$ and Δl.

Feature Point	*d*_1_/cm	*d*_2_/cm	Δ*l*/cm
1	39.88	42.27	2.21
2	39.74	42.09	2.17
3	39.78	42.12	2.16
4	29.12	30.14	0.83
5	21.04	23.54	2.32
6	21.22	23.78	2.37
7	21.19	23.75	2.37
8	29.77	32.97	3.22
9	29.43	32.76	3.15
10	29.56	32.86	3.12
11	29.40	32.75	3.17
12	29.37	32.75	3.20
13	29.17	32.55	3.20
14	29.25	32.58	3.15

**Table 2:** TP, FP, TN, FN values of the four methods.

Experimental Method	TP	FP	TN	FN
VIDAR	3710	397	118	254
VIDAR + MSER	3856	356	41	226
YOLOv8s	3527	362	289	301
Proposed method	4033	146	168	132

**Table 3:** Results of the four methods.

Experimental Method	mAP/%	Recall/%	Accuracy/%	Precision/%	Time/s
VIDAR	89.3	93.6	85.5	90.3	0.324
VIDAR + MSER	92.7	94.4	87.6	91.5	0.343
YOLOv8s	88.1	92.1	85.2	90.7	0.205
Proposed method	96.7	96.8	93.8	96.5	0.317

**Table 4:** Distance measurement results.

Obstacle	Measuring Distance (m)	Actual Distance (m)	Error (m)
1	4.79	4.88	0.09
2	5.13	5.24	0.11
3	7.60	7.73	0.13
4	8.33	8.44	0.11
5	11.09	11.24	0.15
6	10.10	10.27	0.17
7	12.78	12.97	0.19
8	15.72	15.92	0.20
9	15.90	16.08	0.18
10	18.68	18.89	0.21
11	22.53	22.76	0.23
12	12.19	12.34	0.15
13	13.50	13.64	0.14
14	14.58	14.66	0.18
15	9.50	9.65	0.15
16	12.98	12.12	0.14

## Data Availability

Data are contained within the article.
